# Gene expression imputation identifies candidate genes and susceptibility loci associated with cutaneous squamous cell carcinoma

**DOI:** 10.1038/s41467-018-06149-6

**Published:** 2018-10-15

**Authors:** Nilah M. Ioannidis, Wei Wang, Nicholas A. Furlotte, David A. Hinds, 23andMe Research Team, Michelle Agee, Babak Alipanahi, Adam Auton, Robert K. Bell, Katarzyna Bryc, Sarah L. Elson, Pierre Fontanillas, Karen E. Huber, Aaron Kleinman, Nadia K. Litterman, Jennifer C. McCreight, Matthew H. McIntyre, Joanna L. Mountain, Elizabeth S. Noblin, Carrie A. M. Northover, Steven J. Pitts, J. Fah Sathirapongsasuti, Olga V. Sazonova, Janie F. Shelton, Suyash Shringarpure, Chao Tian, Joyce Y. Tung, Vladimir Vacic, Catherine H. Wilson, Carlos D. Bustamante, Eric Jorgenson, Maryam M. Asgari, Alice S. Whittemore

**Affiliations:** 1 Department of Biomedical Data Science, Stanford University School of Medicine, Stanford, CA 94305 USA; 2 Department of Health Research and Policy, Stanford University School of Medicine, Stanford, CA 94305 USA; 3 Department of Genetics, Stanford University School of Medicine, Stanford, California, 94305 USA; 4 23andMe, Inc., Mountain View, CA 94041 USA; 5 Division of Research, Kaiser Permanente Northern California, Oakland, CA 94612 USA; 6 Department of Dermatology, Massachusetts General Hospital, Boston, MA 02114 USA; 8 23andMe, Inc., Mountain View, CA 94041 USA

## Abstract

Cutaneous squamous cell carcinoma (cSCC) is a common skin cancer with genetic susceptibility loci identified in recent genome-wide association studies (GWAS). Transcriptome-wide association studies (TWAS) using imputed gene expression levels can identify additional gene-level associations. Here we impute gene expression levels in 6891 cSCC cases and 54,566 controls in the Kaiser Permanente Genetic Epidemiology Research in Adult Health and Aging (GERA) cohort and 25,558 self-reported cSCC cases and 673,788 controls from 23andMe. In a discovery-validation study, we identify 19 loci containing 33 genes whose imputed expression levels are associated with cSCC at false discovery rate < 10% in the GERA cohort and validate 15 of these candidate genes at Bonferroni significance in the 23andMe dataset, including eight genes in five novel susceptibility loci and seven genes in four previously associated loci. These results suggest genetic mechanisms contributing to cSCC risk and illustrate advantages and disadvantages of TWAS as a supplement to traditional GWAS analyses.

## Abstract (web summary)

Genetic loci linked to susceptibility for the common skin cancer cutaneous squamous cell carcinoma (cSCC) have been identified by genome wide association studies (GWAS). Here, the authors impute gene expression levels from GWAS data to perform a transcriptome wide association study (TWAS), identifying five novel genetic loci linked to cSCC susceptibility.

## Introduction

Cutaneous squamous cell carcinoma (cSCC) is a common and costly form of skin cancer, particularly in individuals of European ancestry^1^. cSCC risk increases with age, fair skin pigmentation, exposure to ultraviolet (UV) radiation, and immunosuppression^2,3^. Three recent genome-wide association studies (GWAS) have identified genetic susceptibility loci for cSCC, including pigmentation-related and non-pigmentation-related loci^4–6^. As a supplement to traditional GWAS analyses, recent methods have been proposed to carry out transcriptome-wide association studies (TWAS) using imputed gene expression levels in GWAS subjects^7,8^. Here we applied TWAS methods to discover gene expression associations with cSCC and compare them to previous cSCC GWAS results.

We previously conducted a GWAS in 7701 cSCC cases and 60,186 non-cSCC controls from among the non-Hispanic white members of the Genetic Epidemiology Research in Adult Health and Aging (GERA) cohort in the Kaiser Permanente Northern California healthcare system^4^. We identified ten loci containing single nucleotide polymorphisms (SNPs) whose dosages were associated with cSCC at genome-wide significance, including six loci (5p13, 6p25, 11q14, 15q13, 16q24 and 20q11) containing genes involved in the pigmentation pathway that regulates the synthesis of melanin^9^ and four additional susceptibility loci (3p13, 3q28, 6p21 and 9p22), including the HLA class II gene locus at 6p21 that encodes the major histocompatibility complex (MHC) class II proteins. An independent cSCC GWAS was performed in 6579 self-reported cSCC cases and 280,558 self-reported non-cSCC controls consented for research with 23andMe, Inc., a personal genetics company, and validated in 825 cSCC cases and 11,518 non-cSCC controls from the Nurses’ Health Study (NHS) and the Health Professionals Follow-Up Study (HPFS)^5^. This GWAS identified 11 loci containing SNPs associated with cSCC at genome-wide significance, including the six pigmentation loci and 9p22 from the Kaiser GWAS, as well as four additional susceptibility loci (2p22, 7p21, 9q34 and 11q23). Finally, a smaller GWAS was performed in 745 cSCC cases and 12,805 non-cSCC controls from NHS, HPFS, and two Rotterdam Study cohorts and validated in 531 independent cSCC cases and 551 independent non-cSCC controls from NHS and HPFS^6^. This GWAS identified five cSCC-associated SNPs in five loci (5q12, 6q26, 8q24, 16q24 and 20q11), although only the SNP at 16q24 was replicated in the Kaiser GERA cohort^10^.

These three cSCC studies all used the standard GWAS approach of evaluating associations between case/control status and dosages at individual germline SNPs. However, the causal genes involved in the observed associations at GWAS susceptibility loci are often unclear. TWAS methods have been proposed to directly identify trait associations with imputed expression levels of individual genes using multiple expression-associated germline SNPs^7,8^. The prediXcan method^7^, for example, trains linear regression models to impute tissue-specific expression levels of individual genes as weighted combinations of dosages at nearby SNPs with elastic net regularization^11^ in a training dataset containing both genotype and gene expression measurements in the same individuals. The Genotype-Tissue Expression (GTEx) Consortium^12,13^ V6p dataset, for example, contains genotypes and RNA sequencing expression data from 44 different tissue types that can be used to train prediXcan regression models to predict the germline genetically-regulated component of the tissue-specific expression levels of individual genes. These prediXcan models are then used to impute tissue-specific gene expression levels for individuals in a separate GWAS dataset, where each gene’s tissue-specific imputed expression level is tested for association with the trait of interest. By pooling information across multiple SNPs in this biologically motivated way, similar to weighted burden tests^14,15^, prediXcan and other TWAS approaches can identify susceptibility loci that were missed in previous GWAS analyses and also suggest candidate causal genes. However, TWAS approaches still suffer from challenges in interpreting causality, such as correlated imputed gene expression levels for nearby genes in the same locus^16^.

Here we use prediXcan models to impute gene expression levels in cSCC cases and controls from the Kaiser GERA cohort and 23andMe research participants and discover novel gene expression associations with cSCC. We compare these TWAS findings to previous GWAS results in the same datasets, describe the associated genes and their potential roles in cSCC pathogenesis, and discuss the strengths and limitations of gene expression imputation and TWAS as highlighted in our study.

## Results

### Discovery and validation in the GERA and 23andMe datasets

We conducted a two-phase discovery and validation study to test for gene expression level associations with cSCC case/control status, using the Kaiser GERA cohort for discovery and the 23andMe research participant dataset for validation, and using prediXcan tissue-specific gene expression imputation models trained on GTEx expression data. Because of previous evidence that TWAS associations in non-disease-relevant tissues are often non-causal^16^, we limited our analysis to four disease-relevant tissue types: the two types of skin tissue available in GTEx (sun-exposed lower leg skin and non-sun-exposed suprapubic skin), as well as whole blood and lymphocyte cell lines (LCLs) based on evidence of immune involvement in cSCC risk^17,18^. Note that the skin tissue expression training data come from bulk skin tissue and are not broken down into specific cell types, such as keratinocytes.

In the Kaiser GERA discovery phase of our study, we identified a total of 33 genes in 19 loci (Supplementary Tables 1–4) whose imputed expression levels in one or more of the four tested tissue types were associated with cSCC at a false discovery rate (FDR) less than 10%. We tested these candidate genes (a total of 50 tissue-specific expression models) for association with cSCC in the 23andMe dataset, and 15 genes in nine loci (Table 1) were validated at a Bonferroni-corrected significance threshold (*P* < 0.001), including eight genes in five novel cSCC susceptibility loci. These validated genes are discussed in detail below. An additional six candidate genes were associated with cSCC in the 23andMe dataset at a more lenient FDR < 10% threshold and are discussed as suggestive associations below.

**Table 1:** Genes associated with cSCC in the discovery-validation TWAS

			PrediXcan	Kaiser GERA analysis			23andMe analysis		
Locus	Gene	Tissue	*R* ^2^	*P*-value	Beta	Beta SE	*P*-value	Beta	Beta SE
**A. Novel loci**									
1q21	*CTSS*	LCLs	0.397	9.68E-06	−0.106	0.024	4.85E-08	−0.065	0.012
	*HORMAD1*	Skin (sun-exp)	0.541	8.59E-05	−0.086	0.022	4.68E-04	−0.038	0.011
	*GOLPH3L*	Skin (sun-exp)	0.446	8.83E-05	−0.094	0.024	5.09E-04	−0.041	0.012
	*ANXA9*	Skin (sun-exp)	0.203	5.12E-04	−0.111	0.032	7.51E-04	−0.053	0.016
2q33	*CASP8*	Skin (sun-exp)	0.413	1.85E-04	−0.097	0.026	1.12E-08	−0.073	0.013
		Skin (non-sun-exp)	0.401	2.58E-04	−0.101	0.028	1.60E-07	−0.071	0.014
6q23	*AHI1*	Skin (sun-exp)	0.493	4.79E-04	0.077	0.022	1.65E-05	0.046	0.011
		Skin (non-sun-exp)	0.473	7.25E-04	0.076	0.022	1.74E-05	0.047	0.011
		LCLs	0.276	1.24E-03	0.085	0.026	2.56E-04	0.046	0.013
12q23	*HAL*	Skin (sun-exp)	0.439	9.98E-04	−0.079	0.024	6.83E-08	−0.064	0.012
		Skin (non-sun-exp)	0.314	1.53E-03	−0.085	0.027	5.63E-07	−0.066	0.013
17q21	*ORMDL3*	LCLs	0.577	2.50E-04	0.081	0.022	9.49E-04	0.036	0.011
**B. Previously associated loci**									
6p21	*HLA-DOB*	Whole blood	0.439	3.77E-04	−0.079	0.022	3.38E-06	−0.051	0.011
	*SKIV2L*	Whole blood	0.259	8.20E-06	−0.143	0.032	3.58E-04	−0.056	0.016
	*HLA-DRB5*	Whole blood	0.592	4.03E-05	−0.081	0.020	9.98E-04	−0.037	0.011
15q13	*HERC2*	Whole blood	0.357	1.52E-04	−0.106	0.028	2.69E-12	−0.097	0.014
16q24	*CDK10*	Skin (non-sun-exp)	0.383	5.57E-34	−0.281	0.023	5.02E-81	−0.209	0.011
		Skin (sun-exp)	0.396	2.02E-23	−0.264	0.026	4.77E-58	−0.212	0.013
		LCLs	0.208	1.71E-22	−0.295	0.030	6.58E-63	−0.245	0.015
		Whole blood	0.423	9.09E-22	−0.213	0.022	1.14E-60	−0.176	0.011
	*FANCA*	LCLs	0.207	7.19E-11	−0.238	0.037	4.12E-31	−0.201	0.017
20q11	*FAM83C*	Skin (non-sun-exp)	0.215	2.80E-05	0.164	0.039	4.05E-13	0.133	0.018

Results from the TWAS discovery phase (Kaiser GERA analysis) and validation phase (23andMe analysis) are shown for all validated genes associated with cSCC.

*R*^2^, squared correlation coefficient for the prediXcan imputation model; *P*-value, from Wald test; Beta SE, standard error in effect size (beta); exp, exposed

### Novel cSCC susceptibility loci

Eight of the cSCC-associated genes identified in our discovery-validation study were located in five previously unidentified cSCC susceptibility loci (Table 1).

At 1q21, imputed expression levels of four genes were negatively associated with cSCC: *CTSS* (cathepsin S) imputed in LCLs, and *HORMAD1* (HORMA domain containing 1), *GOLPH3L* (golgi phosphoprotein 3 like) and *ANXA9* (annexin A9) imputed in sun-exposed skin. The imputed expression levels of these four genes were correlated with one another, with Pearson correlation coefficients ranging from 0.70 to 0.99 among the individuals in the Kaiser GERA cohort (Supplementary Table 5). There was evidence for only one independent association in this region, as the other genes lost significance after adjusting for imputed expression levels of *CTSS* (Supplementary Table 6), which had the strongest individual association. Although none of the individual SNPs in this region met genome-wide significance in the original Kaiser cSCC GWAS, the broad peak in individual-SNP association *P*-values (Supplementary Fig. 1) and the correlation between imputed gene expression levels make it difficult to identify the causal gene at this locus that is driving the observed associations. Several of the associated genes identified in the TWAS analysis have plausible mechanisms of involvement in cSCC. CTSS is a lysosomal cysteine proteinase whose overexpression is linked to tumor progression and angiogenesis in several cancers^19^; however, this observation is inconsistent with the negative direction of association between its imputed expression levels and risk of cSCC. On the other hand, CTSS is also involved in loading antigenic peptides for presentation on MHC class II complexes by degrading the invariant chain that blocks the peptide binding cleft^19^, and immune regulation is known to play a role in cSCC risk^17,18^. GOLPH3L has a regulatory role in Golgi trafficking and is an antagonist of GOLPH3^20^, an oncogene that has been observed at high copy number in several cancers, including melanoma^21^. This antagonist role of GOLPH3L is consistent with the observed negative association of its imputed expression levels with cSCC. HORMAD1 mediates chromosomal recombination during meiosis and is overexpressed in several cancers including melanoma^22,23^, inconsistent with its observed negative cSCC association. Finally, ANXA9 is a calcium and phospholipid binding protein that contains a SNP strongly associated with melanoma^24^, although the mechanism is not yet understood. Further studies are needed to clarify the causal gene(s) and mechanisms of association with cSCC in this region.

At 2q33, imputed expression levels of *CASP8* (caspase 8) in both sun-exposed and non-sun-exposed skin were negatively associated with cSCC. Variants at this locus have been previously associated with esophageal SCC^25^ and cutaneous basal cell carcinoma (cBCC)^26^. In the original Kaiser cSCC GWAS, however, none of the individual SNPs in this region had association *P*-values less than 10^−3^ (Supplementary Fig. 2). CASP8 is a cysteine protease that plays a key role in apoptosis, including apoptosis in response to UV radiation^27,28^. Reduced apoptosis of UV-damaged cells could explain the observed increased risk of cSCC among individuals with lower imputed expression levels of *CASP8*.

At 6q23, imputed expression levels of *AHI1* (Abelson helper integration site 1) in sun-exposed skin, non-sun-exposed skin, and LCLs were positively associated with cSCC. Several of the individual SNPs in this region also had association *P*-values just under 10^−3^ in the original Kaiser cSCC GWAS (Supplementary Fig. 3). *AHI1* is an oncogene that is overexpressed in some types of leukemia and lymphoma, and mutations in *AHI1* are also associated with several brain disorders^29^. The AHI1 protein is involved in vesicle trafficking and in the formation of primary non-motile cilia that are present in most human cells^30^. Although potential mechanisms of involvement of AHI1 in cSCC remain to be determined, its status as an oncogene is consistent with its positive association with cSCC in this study.

At 12q23, imputed expression levels of *HAL* (histidine ammonia-lyase) in both sun-exposed and non-sun-exposed skin were negatively associated with cSCC. A few individual SNPs in this region had association *P*-values around 10^−4^ in the original Kaiser cSCC GWAS (Supplementary Fig. 4). A common nonsynonymous polymorphism in *HAL* (rs7297245) was previously shown to modify the effect of sunburn history on risk of cSCC and cBCC^31^, with a greater increase in risk for homozygous individuals who also had four or more lifetime severe sunburns, although this particular SNP was not individually associated with cSCC in the original Kaiser GWAS (*P* = 0.034). HAL catalyzes the formation in skin of urocanic acid (UCA), a photoreceptor that photoisomerizes in response to UV radiation, promoting suppression of the immune system and raising the risk of UV-induced skin cancers^31,32^. This role of HAL and UCA in UV-mediated immunosuppression is inconsistent with its observed negative association with cSCC. However, UCA also has protective effects against UV-induced DNA damage, and histidinemic mice with a spontaneous mutation in HAL experience more DNA damage after UV exposure than wild type mice^33,34^. Thus, greater protection against DNA damage due to higher levels of UCA could explain the observed decreased risk of cSCC among individuals with higher imputed expression levels of *HAL*.

At 17q21, imputed expression levels of *ORMDL3* (ORMDL sphingolipid biosynthesis regulator 3) in LCLs were positively associated with cSCC. A number of individual SNPs in this region had association *P*-values between 10^−4^ and 10^−5^ in the original Kaiser cSCC GWAS (Supplementary Fig. 5). ORMDL3 is an endoplasmic reticulum transmembrane protein that regulates calcium homeostasis and the unfolded protein response^35,36^. Overexpression of ORMDL3 decreases T lymphocyte activation, and variants in *ORMDL3* are associated with asthma and several other immune-mediated inflammatory diseases^36^. Decreased lymphocyte activity could facilitate tumor evasion of the immune system and explain the observed increased risk of cSCC among individuals with higher imputed expression levels of *ORMDL3* in LCLs.

### Associated genes in known cSCC susceptibility loci

Seven of the cSCC-associated genes identified in our discovery-validation study were located in four known cSCC susceptibility loci (Table 1). Here we compare these gene expression associations with the previous GWAS results for individual SNPs in these loci (Table 2).

**Table 2:** Comparison of TWAS genes and GWAS lead SNPs in shared loci

					Adjusted regressions^a^			
Locus	Gene	Tissue	Gene *P*-value	Lead SNP *P*-value	Gene *P*-value	Lead SNP *P*-value	Correlation^b^	Overlapping GWAS SNPs^c^
6p21	*HLA-DOB*	Whole blood	3.77E-04	6.47E-19	1.98E-01	2.05E-16	−0.258	0 / 33
	*SKIV2L*	Whole blood	8.20E-06	6.47E-19	1.11E-01	5.82E-15	−0.332	0 / 29
	*HLA-DRB5*	Whole blood	4.03E-05	6.47E-19	6.32E-01	4.45E-15	−0.511	1 / 24
15q13	*HERC2*	Whole blood	1.52E-04	4.97E-08	8.19E-02	2.03E-05	−0.440	0 / 32
16q24	*CDK10*	Skin (non-sun-exp)	5.57E-34	5.04E-45	2.55E-03	1.72E-14	−0.708	16 / 35
		Skin (sun-exp)	2.02E-23	5.04E-45	1.07E-01	5.83E-24	−0.612	8 / 16
		LCLs	1.71E-22	5.04E-45	3.27E-02	1.23E-25	−0.558	9 / 16
		Whole blood	9.09E-22	5.04E-45	6.39E-02	5.04E-26	−0.564	12 / 32
	*FANCA*	LCLs	7.19E-11	5.04E-45	2.50E-03	1.39E-37	−0.258	5 / 19
20q11	*FAM83C*	Skin (non-sun-exp)	2.80E-05	7.81E-18	3.84E-01	8.54E-14	0.384	0 / 19

Data from the Kaiser GERA cohort comparing the cSCC associated genes located in previous GWAS susceptibility loci with the most significant SNPs (lead SNPs) from the Kaiser GWAS in those loci.

^a^Results of logistic regression of cSCC case/control status using both the imputed expression level of the indicated gene and dosage of the lead GWAS SNP at that locus as predictors, evaluated in the Kaiser GERA cohort (additional covariates included sex, age, and ten ancestry principal components).

^b^Pearson correlation coefficient between the imputed expression level of the indicated gene and dosage of the lead GWAS SNP at that locus, evaluated among individuals in the Kaiser GERA cohort.

^c^Number of SNPs in the indicated gene expression imputation model that met genome-wide significance for association with cSCC in the previous Kaiser GWAS^4^ / total number of SNPs in the imputation model.

*P*-value, from Wald test; exp, exposed

At 6p21, imputed expression levels of three genes were negatively associated with cSCC: *HLA-DOB* (MHC class II, DO beta), *SKIV2L* (Ski2 like RNA helicase), and *HLA-DRB5* (MHC class II, DR beta 5), all imputed in whole blood. Individual SNPs in 6p21 were previously associated with cSCC at genome-wide significance in the Kaiser GWAS^4^ (Supplementary Fig. 6), with the most significant SNP being rs4455710 in *HLA-DQA1*. Association at this locus suggests a role for HLA antigens and immune response in cSCC^4^, consistent with previous findings of elevated cSCC risk in immunocompromised individuals^17^. Although imputed expression levels of the three associated genes were not correlated with one another among the individuals in the Kaiser GERA cohort (Pearson correlation coefficients < 0.1 for all pairs; Supplementary Table 7), all three were moderately correlated with risk allele dosage at rs4455710, with correlation coefficients ranging from –0.26 to –0.51 (Table 2 and Supplementary Table 7). After adjustment for rs4455710 dosage, none of the three gene expression levels remained associated with cSCC (Table 2 and Supplementary Table 8), indicating only one independent association in this region. However, adjusting for all three genes without including rs4455710 resulted in only moderate attenuation of the effect size and *P*-value for each gene (Supplementary Table 8). Interpreting the association signals at this locus is particularly challenging due to poor imputation quality in the highly polymorphic HLA region, and is the subject of a separate analysis of potential causal HLA alleles and haplotypes^37^.

At 15q13, imputed expression levels of *HERC2* (HECT and RLD domain containing E3 ubiquitin protein ligase 2) in whole blood were negatively associated with cSCC. Individual SNPs in *HERC2* and the nearby gene *OCA2* were associated with cSCC in previous GWAS^4,5^ (Supplementary Fig. 7) and have also been associated with pigmentation phenotypes^38–40^. Expression levels of *OCA2* could not be imputed by prediXcan in the relevant tissue types and thus were not tested in our analysis. Imputed expression levels of *HERC2* were moderately negatively correlated (correlation coefficient –0.44; Table 2) with risk allele dosage at the most significantly associated SNP in this locus (rs12916300) from the Kaiser GWAS, which lies in an intron of *HERC2*. After adjustment for rs12916300, imputed expression levels of *HERC2* were no longer associated with cSCC (Table 2). However, the observed negative association of imputed *HERC2* expression with cSCC is consistent with its role in promoting DNA repair after exposure to ionizing radiation^41^.

At 16q24, imputed expression levels of two genes were negatively associated with cSCC: *CDK10* (cyclin dependent kinase 10) imputed in all four tested tissue types, and *FANCA* (Fanconi anemia complementation group A) imputed in LCLs. Individual SNPs in 16q24 were associated with cSCC in all three previous GWAS, with the most significantly associated SNPs being rs4268748 and rs8063761 in *DEF8*^4,6^ (Supplementary Fig. 8) and rs1805007 in *MC1R*^5^. Imputed expression levels of *CDK10* and *FANCA* among the individuals in the Kaiser GERA cohort were moderately negatively correlated with risk allele dosage at the lead Kaiser SNP rs4268748, with correlation coefficients ranging from –0.26 to –0.71 (Table 2 and Supplementary Table 9), although expression levels of these two genes were poorly correlated with one another (Supplementary Table 9). After adjustment for rs4268748, expression levels of *CDK10* in non-sun-exposed skin and *FANCA* in LCLs retained borderline significance for association with cSCC, with nominal association *P*-values of 0.0026 and 0.0025, respectively (Table 2 and Supplementary Table 10). Expression levels of *FANCA* remained strongly associated with cSCC after adjustment for *CDK10* expression (Supplementary Table 10), suggesting that these two genes represent independent cSCC associations. High copy numbers of a region containing *CDK10* have been associated with better survival of patients with oropharyngeal SCC^42^, and *CDK10* also acts as a tumor suppressor in several other cancers^43,44^, consistent with its observed negative association with cSCC. Mutations in *FANCA* cause a recessive disease involving chromosomal instability, defective DNA repair, and predisposition to cancer^45,46^, also consistent with its observed negative association with cSCC. In addition, the nearby gene *MC1R*, identified as a candidate causal gene in previous GWAS^4–6^, is part of the pigmentation pathway regulating the synthesis of melanin. *MC1R* expression levels could not be imputed by prediXcan in either sun-exposed or non-sun-exposed skin, only in LCLs and whole blood. Its imputed expression levels in LCLs, but not whole blood, were strongly associated with cSCC in the Kaiser GERA cohort in a candidate gene analysis (Table 3). However, because these imputed expression levels in LCLs and whole blood were poorly correlated with measured *MCIR* expression levels in the GTEx dataset used to train prediXcan (Table 3), they were not included in our full transcriptome-wide discovery-validation study (see Methods). Because of these complications, it is difficult to determine whether the observed *MC1R* association in LCLs is truly driven by differential expression of *MC1R,* or whether it is more likely driven by one or more individual SNPs in that particular imputation model that are associated with cSCC for other reasons. Expression levels of *DEF8*, the other GWAS candidate gene at this locus, were not associated with cSCC in sun-exposed and non-sun-exposed skin, only in whole blood where the pediXcan model again had poor imputation quality (Table 3). Finally, the previous Kaiser GWAS identified multiple independent associations at this locus, with SNPs rs35063026 in *SPATA33* and rs78703231 in *SPIRE2* retaining significance after adjustment for rs4268748^4^. Expression levels of *SPATA33* could only be imputed in whole blood and were associated with cSCC, but with poor imputation quality (Table 3). Expression levels of *SPIRE2* were moderately well imputed in sun-exposed skin and associated with cSCC at borderline significance (Table 3).

**Table 3:** Results for other candidate genes from previous GWAS

			PrediXcan	Kaiser GERA
Locus	Candidate gene (Ref.)	Tissue	*R* ^2^	*P*-value
2p22	*AC012593.1* (Chahal)	-	-	-
3p13	*FOXP1* (Asgari)	Skin (sun-exp)	0.024	0.074
3q28	*TPRG1* (Asgari)	Skin (sun-exp)	0.071	0.20
		Skin (non-sun-exp)	0.044	0.72
	*TP63* (Asgari)	Skin (non-sun-exp)	0.033	0.44
5p13	*SLC45A2* (Asgari; Chahal)	Whole blood	0.21	0.15
5q12	*ERBIN*/*ERBB2IP* (Siiskonen)	Skin (sun-exp)	0.0077	0.16
6p21	*HLA-DQA1* (Asgari)	LCLs	0.71	0.59
		Whole blood	0.70	0.081
	*HLA-DQB1* (Asgari)	LCLs	0.68	1.4E-04
		Whole blood	0.74	1.7E-03
6p25	*IRF4* (Asgari; Chahal)	LCLs	0.076	1.6E-82
		Whole blood	0.036	8.9E-81
6q26	*PRKN*/*PARK2* (Siiskonen)	-	-	-
7p21	*AHR* (Chahal)	Skin (non-sun-exp)	0.13	0.90
		Whole blood	0.063	0.049
8q24	*ST3GAL1* (Siiskonen)	Skin (non-sun-exp)	0.026	0.95
		LCLs	0.047	0.078
		Whole blood	0.071	0.24
9p22	*BNC2* (Asgari; Chahal)	Skin (sun-exp)	0.023	0.57
		Whole blood	0.026	7.0E-06
	*CNTLN* (Asgari; Chahal)	Skin (sun-exp)	0.026	0.48
		Whole blood	0.0077	0.12
9q34	*SEC16A* (Chahal)	Whole blood	0.29	9.1E-03
11q14	*TYR* (Asgari; Chahal)	-	-	-
11q23	*CADM1* (Chahal)	-	-	-
	*BUD13* (Chahal)	Whole blood	0.045	0.68
15q13	*OCA2* (Asgari; Chahal)	-	-	-
16q24	*DEF8* (Asgari; Siiskonen)	Skin (sun-exp)	0.28	0.74
		Skin (non-sun-exp)	0.16	0.73
		Whole blood	0.016	1.3E-14
	*MC1R* (Asgari; Chahal)	LCLs	0.11	1.2E-19
		Whole blood	0.095	0.76
	*SPATA33* (Asgari)	Whole blood	0.047	1.9E-20
	*SPIRE2* (Asgari)	Skin (sun-exp)	0.31	0.012
		Skin (non-sun-exp)	0.11	0.097
		Whole blood	0.16	0.38
20q11	*RALY* (Asgari; Chahal)	-	-	-
	*ASIP* (Asgari; Chahal)	Skin (sun-exp)	0.047	4.8E-04
	*SRC* (Siiskonen)	-	-	-

Imputation and association results in the Kaiser GERA cohort for all genes discussed as candidate causal genes in previous cSCC GWAS publications, except those already shown in Tables 1 and 2. For each gene, results are presented for all tissues in which the expression level could be imputed using prediXcan; genes whose expression could not be imputed in any of the four tested tissue types are indicated with (-).

Ref., reference GWAS publication; *R*^2^, squared correlation coefficient for the prediXcan imputation model; *P*-value, from Wald test; exp, exposed; Asgari, Asgari et al^4^; Chahal, Chahal et al^5^; Siiskonen, Siiskonen et al.^6^

At 20q11, imputed expression levels of *FAM83C* (family with sequence similarity 83 member C) in non-sun-exposed skin were positively associated with cSCC. Individual SNPs in 20q11 were associated with cSCC in all three previous GWAS, with the most significant SNPs being rs6059655 in *RALY*^4,5^ (Supplementary Fig. 9) and rs754626 in *SRC*^6^. Expression levels of *RALY* and *SRC* could not be imputed by prediXcan in the relevant tissue types and thus were not tested in our analysis. Imputed expression levels of *FAM83C* were moderately correlated with risk allele dosage at the lead Kaiser SNP rs6059655 (correlation coefficient 0.38; Table 2), and imputed expression levels of *FAM83C* were no longer associated with cSCC after adjustment for rs6059655 (Table 2). However, *FAM83C* is an oncogene that activates MAPK signaling and promotes cancer progression^47^, consistent with its observed positive association with cSCC. This locus also contains the pigmentation-related gene *ASIP*, considered a candidate causal gene in previous GWAS^4,5^. Expression levels of *ASIP* could only be imputed in sun-exposed skin, where they were borderline associated with cSCC in the Kaiser GERA cohort (Table 3). However, these imputed *ASIP* expression levels were poorly correlated with measured *ASIP* expression levels in the GTEx dataset used to train prediXcan (Table 3), again making it difficult to determine whether the observed association is driven by differential expression of *ASIP* or by other causal mechanisms tagged by one or more of the individual SNPs included in the imputation model.

### Suggestive associations

Six additional genes in the validation phase of our study passed a significance threshold of FDR < 10% and were considered suggestive associations, including three genes in two new loci (2q14 and 3q25). At 2q14, imputed expression levels of *ACTR3* (ARP3 actin related protein 3 homolog) in sun-exposed skin and *SLC35F5* (solute carrier family 35 member F5) in non-sun-exposed skin were positively associated with cSCC (Supplementary Tables 1 and 2). *ACTR3*, also known as *ARP3*, encodes a protein involved in actin polymerization, essential to cell motility and metastasis of cancer cells. Expression of ARP3 in tumor cells has been associated with poor prognosis of several cancers, including SCC of the gallbladder^48^. *SLC35F5*, at the same locus, encodes a membrane transporter that has not been well characterized. At 3q25, imputed expression levels of *LEKR1* (leucine, glutamate and lysine rich 1) in LCLs were positively associated with cSCC (Supplementary Table 3). The protein encoded by *LEKR1* has also not been well characterized, but a missense variant in *LEKR1* was previously associated with epithelial ovarian cancer^49^. The remaining three genes with suggestive associations were located in previously-discussed loci. At 6p21, imputed expression levels of *POU5F1* (POU class 5 homeobox 1) in non-sun-exposed skin were negatively associated with cSCC (Supplementary Table 2); at 17q21, imputed expression levels of *GSDMB* (gasdermin B) in whole blood and LCLs were positively associated with cSCC (Supplementary Tables 3 and 4); and at 20q11, imputed expression levels of *MAPRE1* (microtubule associated protein RP/EB family member 1) in sun-exposed and non-sun-exposed skin were positively associated with cSCC (Supplementary Tables 1 and 2). These associations provide additional candidate causal genes at their respective loci, although with lower significance than those discussed previously.

Finally, at 10q25, imputed expression levels of *GSTO2* (glutathione S-transferase omega 2) in non-sun-exposed skin were negatively associated with cSCC at a nominal *P*-value that passed transcriptome-wide Bonferroni significance in the Kaiser GERA cohort (Supplementary Table 11), although this association was not validated in the 23andMe dataset (Supplementary Table 2). Variants in *GSTO2* have been previously associated with cancer risk^50^, presumably due to its role in metabolizing and detoxifying chemical carcinogens and reactive oxygen species. This protective role is consistent with the observed decreased risk of cSCC among individuals with higher imputed expression levels of *GSTO2* in the Kaiser GERA cohort.

### Additional candidate genes from previous cSCC GWAS

We also examined the expression imputation and cSCC association results for all other genes that had been suggested as candidate causal genes at cSCC susceptibility loci in previous GWAS analyses (Table 3). Many of these candidate genes had expression levels that were poorly imputed by the prediXcan models, with low squared correlation coefficients between imputed and observed expression levels in the GTEx training dataset (Table 3), making their association results difficult to interpret. A few previous candidate genes had strong evidence of association with cSCC in one or more tested tissue types despite being poorly imputed, including *IRF4* at 6p25, *BNC2* at 9p22, and *DEF8*, *MC1R*, and *SPATA33* at 16q24 (Table 3). These associations, in light of the poor expression imputation quality, suggest that the linear regression models used to impute the expression levels of these genes may contain SNPs that are strongly associated with cSCC through mechanisms other than differential gene expression.

## Discussion

We imputed the germline genetically-regulated component of gene expression levels in skin tissue, whole blood, and LCLs as linear combinations of genotype dosages at nearby expression-associated SNPs and tested association of these imputed expression levels with cSCC in two independent GWAS datasets. We performed an initial discovery TWAS in the Kaiser GERA cohort and passed the resulting candidate genes on to validation in 23andMe research participants. We validated a total of 15 cSCC-associated genes at Bonferroni significance, including eight genes in five novel cSCC susceptibility loci: *CTSS*, *HORMAD1*, *GOLPH3L* and *ANXA9* at 1q21, *CASP8* at 2q33, *AHI1* at 6q23, *HAL* at 12q23, and *ORMDL3* at 17q21. The identification of these novel susceptibility loci reflects both the reduced multiple hypothesis testing burden of TWAS relative to GWAS and the combination of small effects across multiple SNPs in the linear regression models for gene expression. Most of these genes have plausible biological mechanisms of involvement in cSCC that are consistent with the observed direction of effect on cSCC risk for individuals with higher versus lower imputed expression levels. However, as these results are based solely on statistical associations, experimental validation is needed to confirm differential expression of these genes in cSCC cases and controls and to clarify their potential roles in cSCC pathogenesis, particularly at the 1q21 locus where we found multiple associated genes with plausible causal mechanisms but only one independent association.

Our validated cSCC associations also included seven genes in four loci containing SNPs that had been associated with cSCC in previous GWAS: *HLA-DOB*, *SKIV2L* and *HLA-DRB5* at 6p21, *HERC2* at 15q13, *CDK10* and *FANCA* at 16q24, and *FAM83C* at 20q11. In these loci, the expression associations suggest new candidate causal genes that may be involved in cSCC pathogenesis. For example, both *FANCA* and *FAM83C* are candidate causal genes whose biological functions are consistent with their observed direction of association with cSCC risk, but that had not been considered candidate genes based on the results of earlier GWAS analyses. However, most of the observed gene expression associations in these previously associated loci did not persist after adjustment for the most significant individual GWAS SNP (Table 2), suggesting that there are other factors contributing to cSCC association at these loci beyond differential gene expression. Distinguishing between TWAS associations that reflect causal differential expression mechanisms and those that are partially tagging other causal associations at the same locus is an important goal of both future methods development and follow-up experimental studies.

Interpreting TWAS findings is also complicated by the fact that many gene expression levels are poorly imputed in many disease-relevant tissues using currently available methods for expression imputation. In this study, for example, the expression levels of several candidate genes related to skin pigmentation (e.g. *OCA2* at 15q13, *MC1R* at 16q24, and *ASIP* at 20q11) were poorly imputed (Table 3), making it difficult to draw conclusions about the most likely causal genes at these loci. Some of these genes may have a large environmental component to their expression regulation, while others may require more complex statistical imputation models to capture the full range of genetic effects on their expression. In addition, the expression data used to train the skin and whole blood models came from bulk tissue, rather than individual cell types; thus cell type heterogeneity is an additional source of variation. Some cSCC GWAS candidate genes with poorly imputed expression levels did have evidence of association with cSCC in at least one tissue, including *IRF4*, *BNC2*, *DEF8*, *MCIR*, *SPATA33* and *ASIP* (Table 3). However, the poor performance of their expression imputation models in the GTEx training set suggests that some individual SNPs used in the models may be associated with cSCC through other causal mechanisms not related to differential expression of these particular genes. Because of the difficulty in interpreting associations for poorly imputed genes, in our transcriptome-wide discovery and validation study we considered only genes with squared correlation coefficients between imputed and observed expression levels of *R*^2^ > 0.2. Improving the accuracy of gene expression imputation in future studies will increase power to detect gene expression associations with clinical traits and enable more robust interpretation of results.

Despite these limitations, our results demonstrate that TWAS approaches provide a valuable supplement to individual-SNP GWAS analyses and can identify additional trait-associated loci and candidate genes. The observed direction of association between imputed gene expression levels and the trait of interest guides hypotheses about the potential biological mechanisms underlying each gene-trait association. There is a clear need for follow-up experimental studies to confirm differential expression of the associated genes in cSCC cases and controls, to test the hypothesized biological mechanisms of involvement in cSCC, and to distinguish between multiple candidate causal genes at individual loci. Nevertheless, the cSCC associations identified in this study aid in selecting candidate genes to be prioritized in experimental studies and improve our understanding of the genetic risk factors for this disease.

## Methods

### Study populations

The discovery set consisted of 6891 cSCC cases and 54,566 non-cSCC controls from the non-Hispanic white Kaiser GERA cohort used for the previous Kaiser GERA cSCC GWAS^4^. The 6891 cSCC cases were those GERA participants with a pathology record consistent with at least one incident cSCC during the period from GERA enrollment to last observation before December 31, 2012. The 54,566 controls had no pathology records consistent with any skin cancer and no reported history of any skin cancer prior to GERA enrollment. Cases and controls were at least 18 years of age and were genotyped using a custom Affymetrix Axiom® array optimized for individuals of European ancestry, imputed to 1000 Genomes Project SNPs^4^. This study was conducted in compliance with all relevant ethical regulations, and all research participants provided informed consent under a study protocol approved by the Institutional Review Board (IRB) of the Kaiser Foundation Research Institute.

The validation set consisted of 25,558 self-reported cSCC cases and 673,788 self-reported non-cSCC controls consented for research with 23andMe, Inc. (Mountain View, CA). All participants provided informed consent and participated in the research online, under a protocol approved by the external Association for the Accreditation of Human Research Protection Program (AAHRPP)-accredited IRB, Ethical and Independent Review Services (E&I Review). Participants were genotyped on one of four 23andMe genotyping chips and imputed to 1000 Genomes Project SNPs^5^. Participants also met the same inclusion criteria that were used for the previous 23andMe cSCC GWAS^5^, with at least 97% European ancestry as determined by local ancestry analysis, between 18 and 79 years of age, and unrelated to another individual in the dataset by more than 700 cM shared regions of identity-by-descent. The 25,558 cSCC cases had indicated a cSCC diagnosis in response to at least one of five relevant survey questions, while the 673,788 controls also responded to at least one of the five questions and indicated no cSCC diagnosis. In particular, the relevant questions were: “Have you ever been diagnosed by a doctor with any of the following common cancers? Squamous cell carcinoma [Yes/No/I don’t know]”; “What type(s) of skin cancer did you have? Please check all that apply. Squamous cell carcinoma [Yes/No]”; “What type of skin cancer or cancers have you been diagnosed with? Please check all that apply. Squamous cell carcinoma [Yes/No]”; “In the last 2 years, have you been newly diagnosed with or started treatment for any of the following conditions? Squamous cell carcinoma [Yes/No]”; “In the last 2 years, have you been newly diagnosed with or newly prescribed treatment for any of the following conditions by a medical professional? Squamous cell carcinoma [Yes/No].”

### Gene expression imputation

We obtained prediXcan regression coefficients for predicting gene expression levels as linear combinations of selected SNP dosages from the prediXcan PredictDB database (predictdb.hakyimlab.org)^7^. In particular, we used models released in 2016 (GTEx-V6p-HapMap-2016-09-08) based on the HapMap SNP set and trained using the Genotype-Tissue Expression (GTEx) Consortium V6p RNA expression dataset^12,13^. We imputed tissue-specific gene expression levels for each GWAS subject using the python scripts provided with prediXcan, focusing on four tissue types that are most likely to have direct relevance to cSCC pathogenesis: sun-exposed (lower leg) skin (7665 genes), non-sun-exposed (suprapubic) skin (5471 genes), whole blood (6588 genes), and LCLs (3441 genes). In the discovery phase of the study, described below, to avoid increasing the multiple hypothesis testing burden by including genes with poorly predicted expression levels, we limited the set of genes tested for cSCC association to those with imputation *R*^2^ > 0.2, where the *R*^2^ value is the cross-validated squared Pearson correlation coefficient between observed and imputed expression levels in the GTEx training dataset used by prediXcan^7^. There were 1857 total genes with *R*^2^ above this threshold in at least one of the four tested tissue types: 941 genes in sun-exposed skin, 755 genes in non-sun-exposed skin, 809 genes in whole blood, and 642 genes in LCLs.

### Association of imputed gene expression levels with cSCC

We used logistic regression, as implemented in the glm function in R^51^, to test for association between cSCC case/control status and each of the imputed tissue-specific gene expression levels described above. For discovery in the Kaiser GERA cohort, each regression model included as predictors the imputed expression level of a given gene, sex, age at first diagnosis for cases and age at GERA enrollment for controls, and the top ten principal components of ancestry^4^. For validation in the 23andMe dataset, each regression model similarly included the imputed gene expression level, sex, age at time of survey question response, and the top ten principal components of ancestry^5^. We obtained the effect size and *P*-value from the Wald test, as implemented in the glm function, for each tested gene expression level and computed false discovery rates (FDRs) using the Benjamini-Hochberg method^52^ implemented in the p.adjust function in R^51^.

### Discovery and validation study design

We performed two phases of analysis to identify genes whose imputed expression levels were associated with risk of cSCC. For discovery, we conducted a transcriptome-wide analysis using the Kaiser GERA cohort and obtained the association *P*-value, effect size, and FDR for each tested tissue-specific gene expression level as described above. The set of genes within each tissue whose imputed expression levels were associated with cSCC at FDR < 10% were considered candidate genes to be tested in the validation phase of the study using the larger 23andMe dataset. In particular, a total of 50 tissue-specific imputation models (corresponding to 33 genes located in 19 genomic loci) were tested in the validation phase (Supplementary Tables 1–4). We used a Bonferroni-corrected significance threshold (*P* < 0.001) as the threshold for validation. For comparison with the previous cSCC GWAS in the Kaiser GERA cohort^4^, Supplementary Table 11 also lists the genes and loci that were transcriptome-wide significant in the Kaiser GERA cohort at Bonferroni-corrected significance in at least one of the tested tissue types.

## Electronic supplementary material

Supplementary Information

## Data Availability

Genotype data for the Kaiser GERA cohort are available from the database of Genotypes and Phenotypes (dbGaP) under accession phs000674.v2.p2. This includes individuals who consented to having their data shared with dbGaP. The complete GERA data are available upon application to the Kaiser Permanente Research Bank (researchbank.kaiserpermanente.org). The summary statistics for association between cSCC and all tested gene expression imputation models in the Kaiser GERA cohort are available from the corresponding authors upon request. Genotype data for the 23andMe research participants have not been deposited in public repositories, as consent for this was not obtained in the study protocol. Summary statistics for association between cSCC and all 50 gene expression imputation models tested in the 23andMe dataset are provided in Supplementary Tables 1–4. Other summary statistics from the 23andMe analysis can be made available to qualified investigators who enter into an agreement with 23andMe that protects participant confidentiality. Interested investigators should email dataset-request@23andme.com for more information.
