# Supplementary material for: Gene expression imputation identifies candidate genes and susceptibility loci associated with cutaneous squamous cell carcinoma
Source: Nat Commun. 2018 Oct 15;9:4264. doi: 10.1038/s41467-018-06149-6 (PMC6189170; doi:10.1038/s41467-018-06149-6)
Supplement: Supplementary file 1 — Supplementary Information [file 41467_2018_6149_MOESM1_ESM.pdf]

SUPPLEMENTARY INFORMATION

Gene expression imputation identifies candidate genes and susceptibility loci associated with risk of cutaneous squamous cell carcinoma

Ioannidis et al.

Supplementary Figure 1

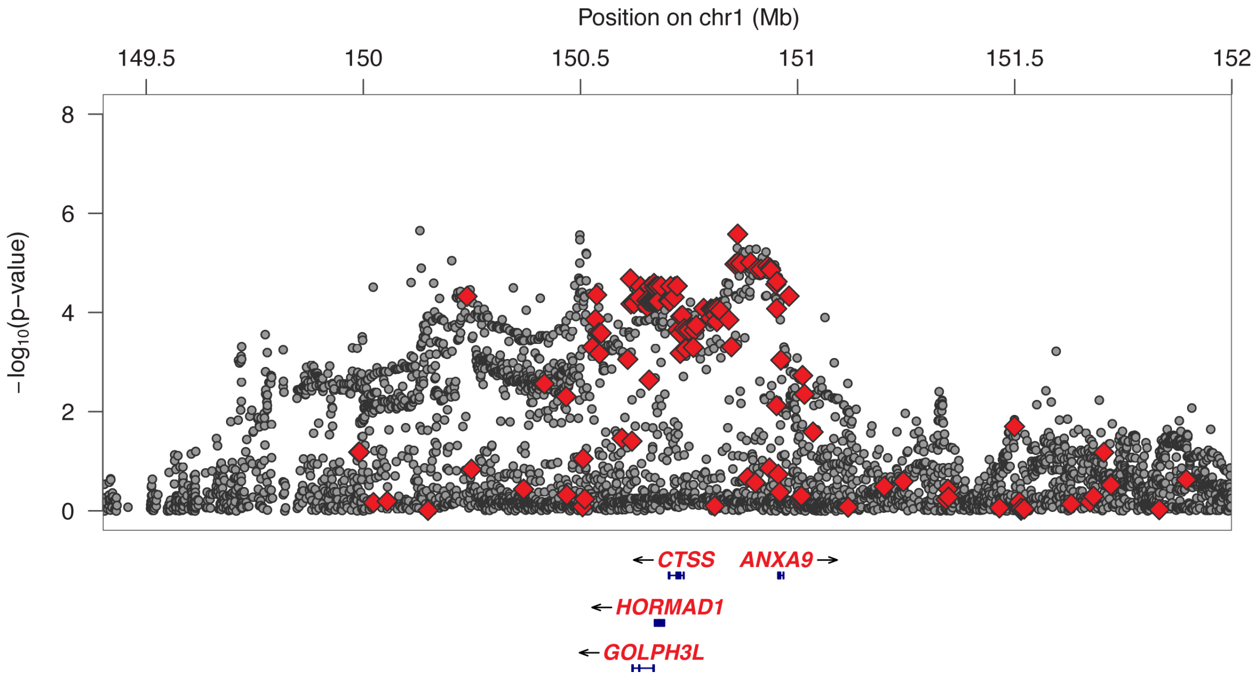

Manhattan plot of individual SNP cSCC associations at the 1q21 locus. Significance levels from the Kaiser cSCC GWAS[1] are plotted for all SNPs in the region (gray circles) and for the subset of SNPs (red diamonds) with nonzero coefficients in one or more of the prediXcan expression models for the associated genes at this locus. The locations of the associated genes (red text) are also shown, with arrows indicating the transcribed strand and ticks indicating exons. Plotting was done using LocusZoom[2].

Supplementary Figure 2

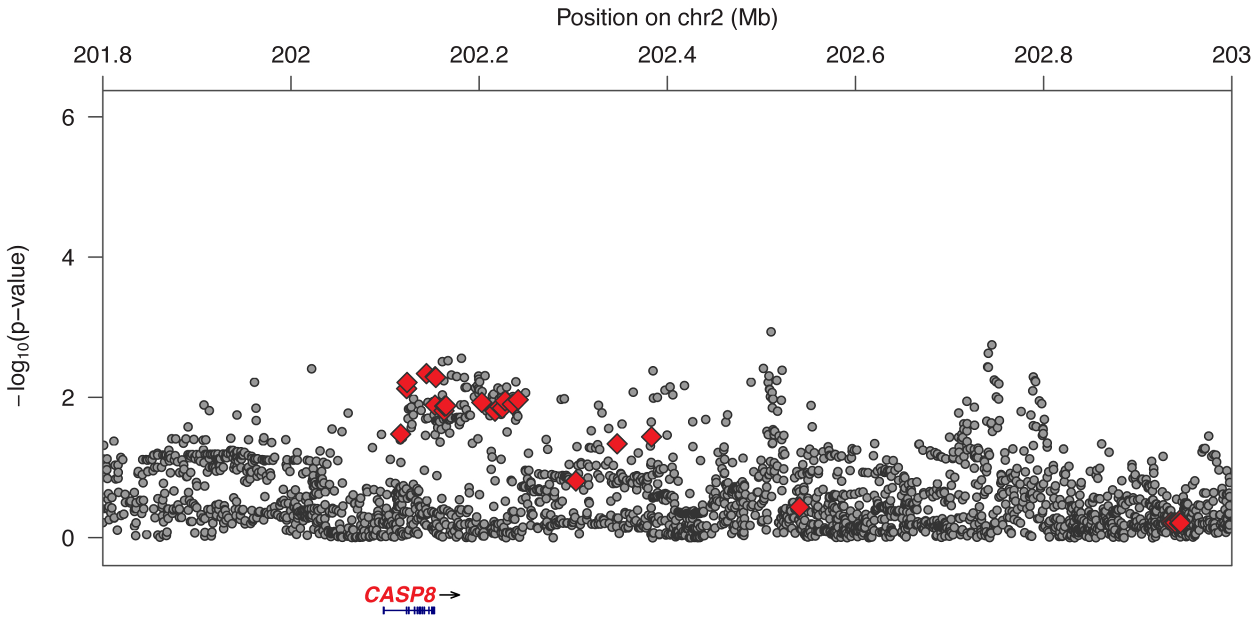

Manhattan plot of individual SNP cSCC associations at the 2q33 locus. Significance levels from the Kaiser cSCC GWAS[1] for all SNPs in the region (gray circles) and the subset of SNPs (red diamonds) with nonzero coefficients in the prediXcan expression models for the associated gene (red text), plotted as in Supplementary Fig. 1.

Supplementary Figure 3

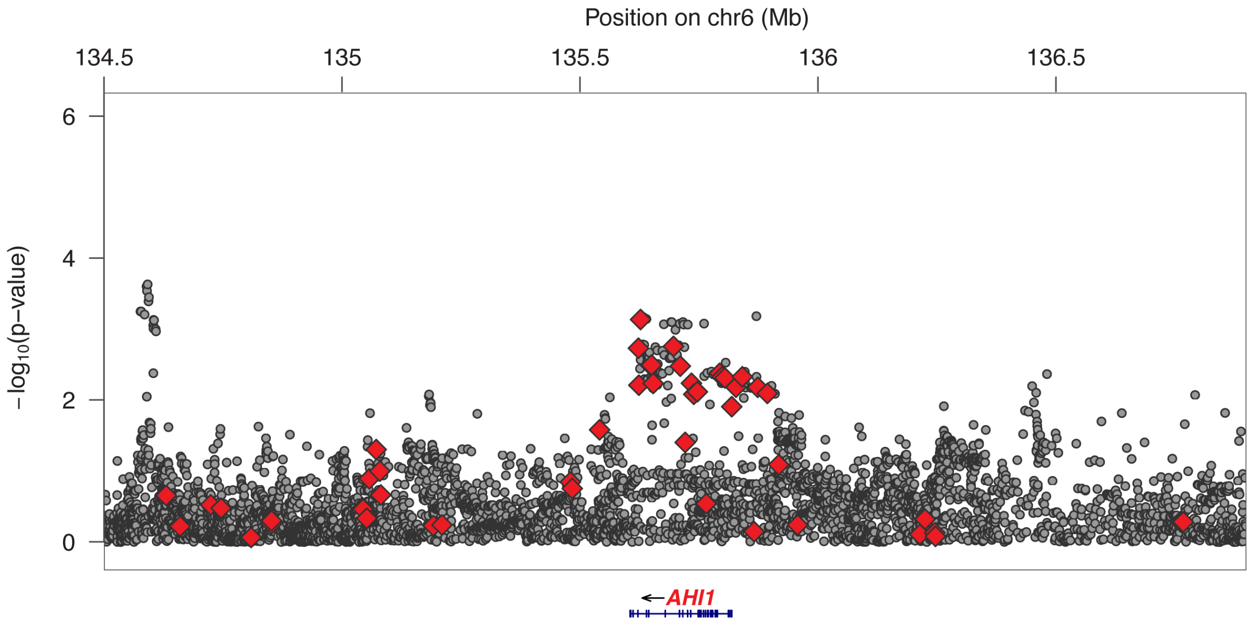

Manhattan plot of individual SNP cSCC associations at the 6q23 locus. Significance levels from the Kaiser cSCC GWAS[1] for all SNPs in the region (gray circles) and the subset of SNPs (red diamonds) with nonzero coefficients in the prediXcan expression models for the associated gene (red text), plotted as in Supplementary Fig. 1.

Supplementary Figure 4

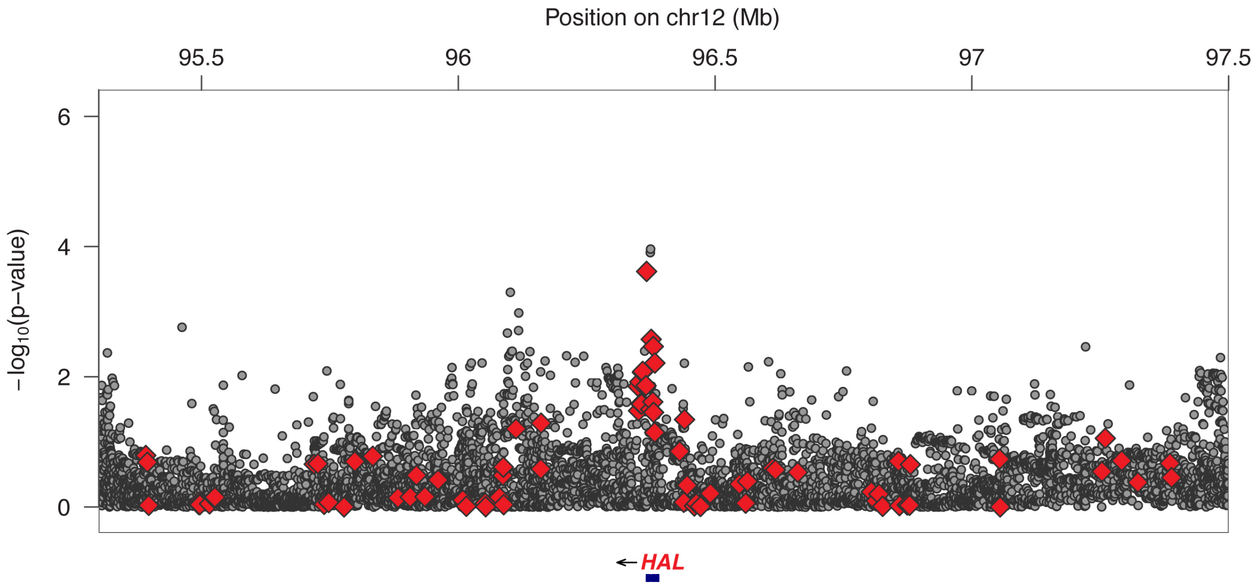

Manhattan plot of individual SNP cSCC associations at the 12q23 locus. Significance levels from the Kaiser cSCC GWAS[1] for all SNPs in the region (gray circles) and the subset of SNPs (red diamonds) with nonzero coefficients in the prediXcan expression models for the associated gene (red text), plotted as in Supplementary Fig. 1.

Supplementary Figure 5

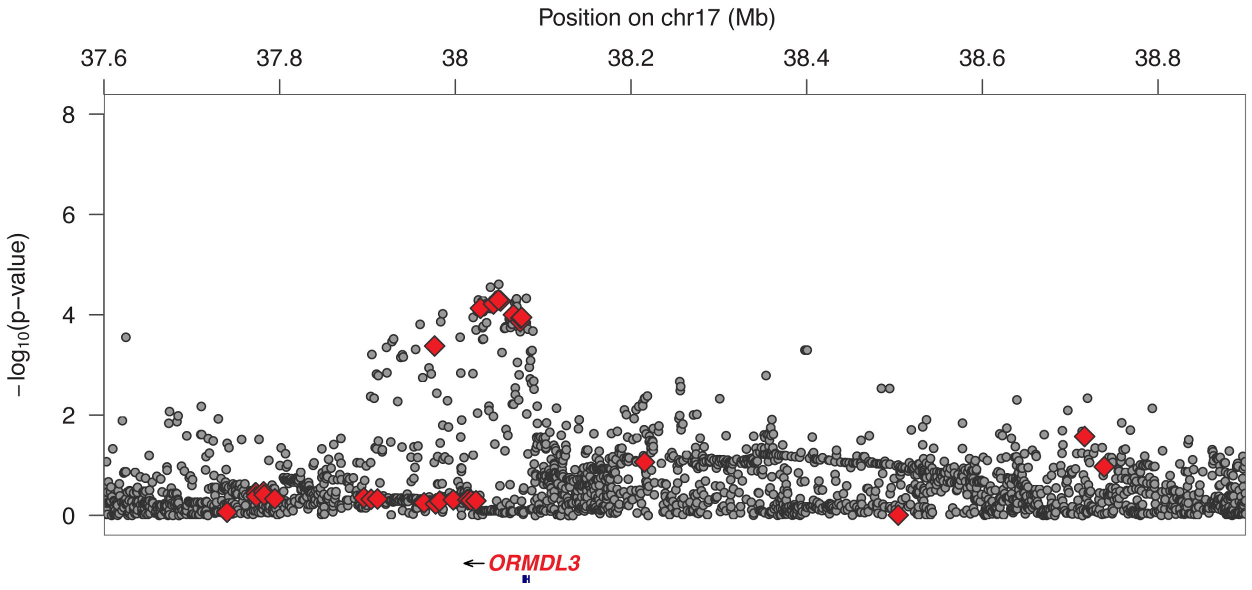

Manhattan plot of individual SNP cSCC associations at the 17q21 locus. Significance levels from the Kaiser cSCC GWAS[1] for all SNPs in the region (gray circles) and the subset of SNPs (red diamonds) with nonzero coefficients in the prediXcan expression model for the associated gene (red text), plotted as in Supplementary Fig. 1.

Supplementary Figure 6

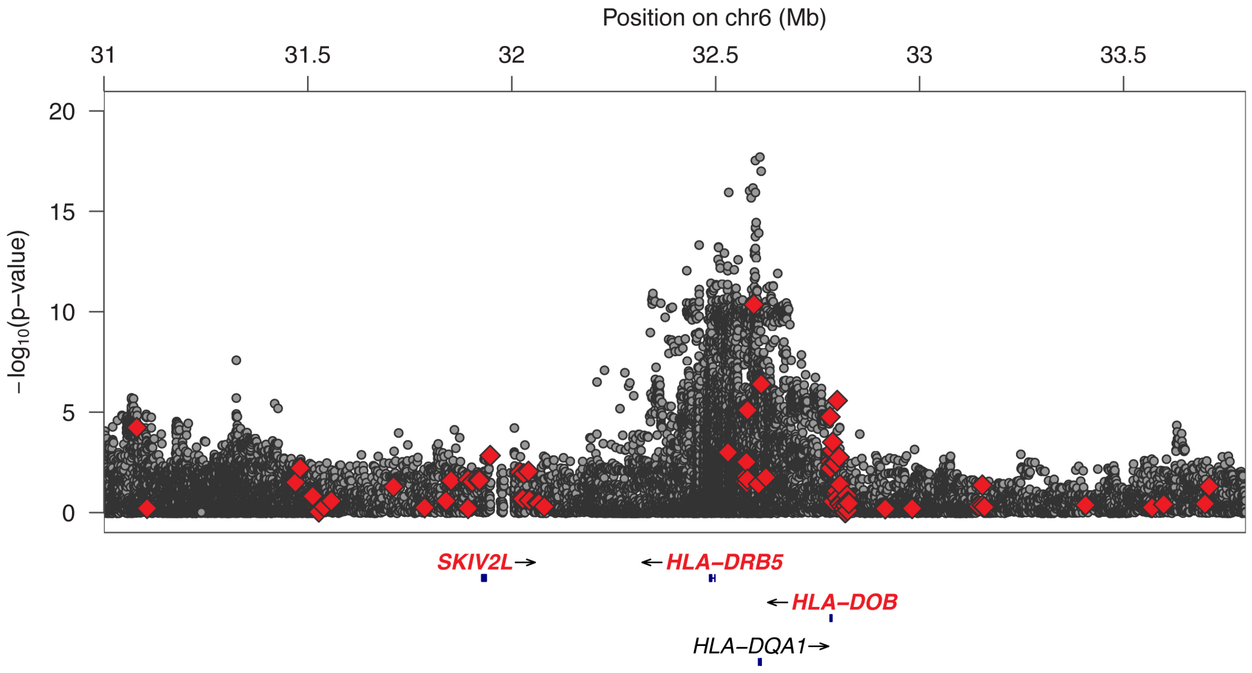

Manhattan plot of individual SNP cSCC associations at the 6p21 locus. Significance levels from the Kaiser cSCC GWAS[1] are plotted for all SNPs in the region (gray circles) and for the subset of SNPs (red diamonds) with nonzero coefficients in one or more of the prediXcan expression models for the associated genes at this locus. The locations of the associated genes (red text) and other candidate genes from the previous GWAS (black text) are also shown, with arrows indicating the transcribed strand and ticks indicating exons. Plotting was done using LocusZoom[2].

Supplementary Figure 7

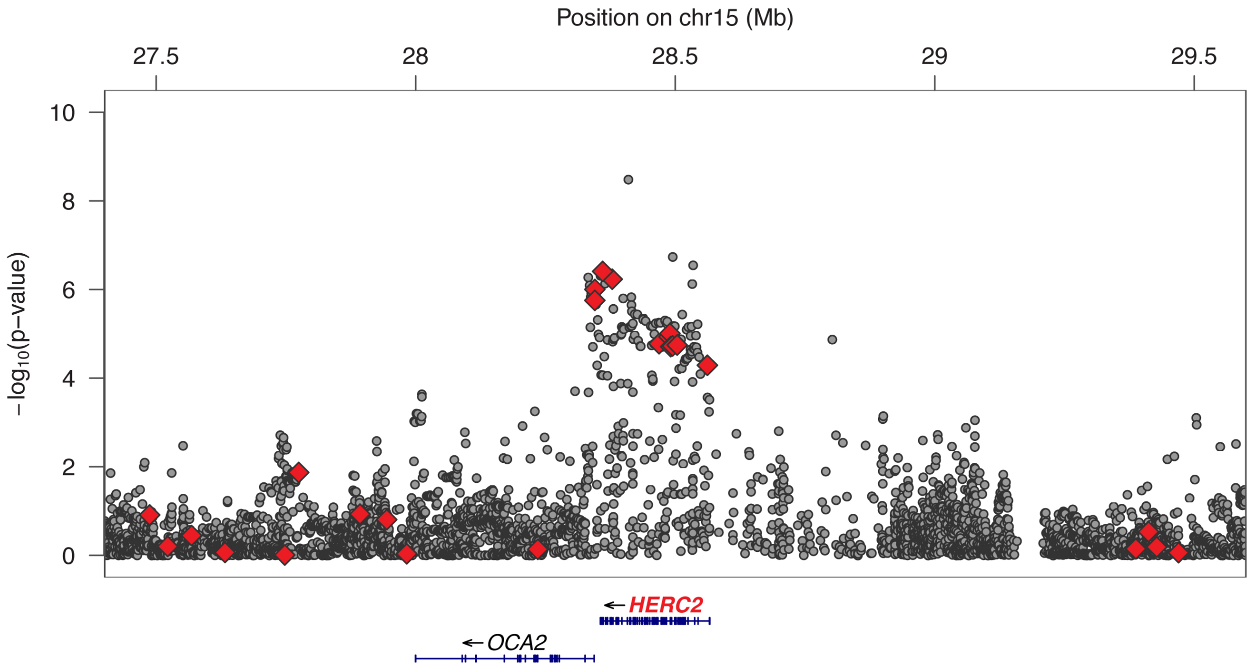

Manhattan plot of individual SNP cSCC associations at the 15q13 locus. Significance levels from the Kaiser cSCC GWAS[1] for all SNPs in the region (gray circles) and the subset of SNPs (red diamonds) with nonzero coefficients in the prediXcan expression model for the associated gene (red text), plotted as in Supplementary Fig. 6.

Supplementary Figure 8

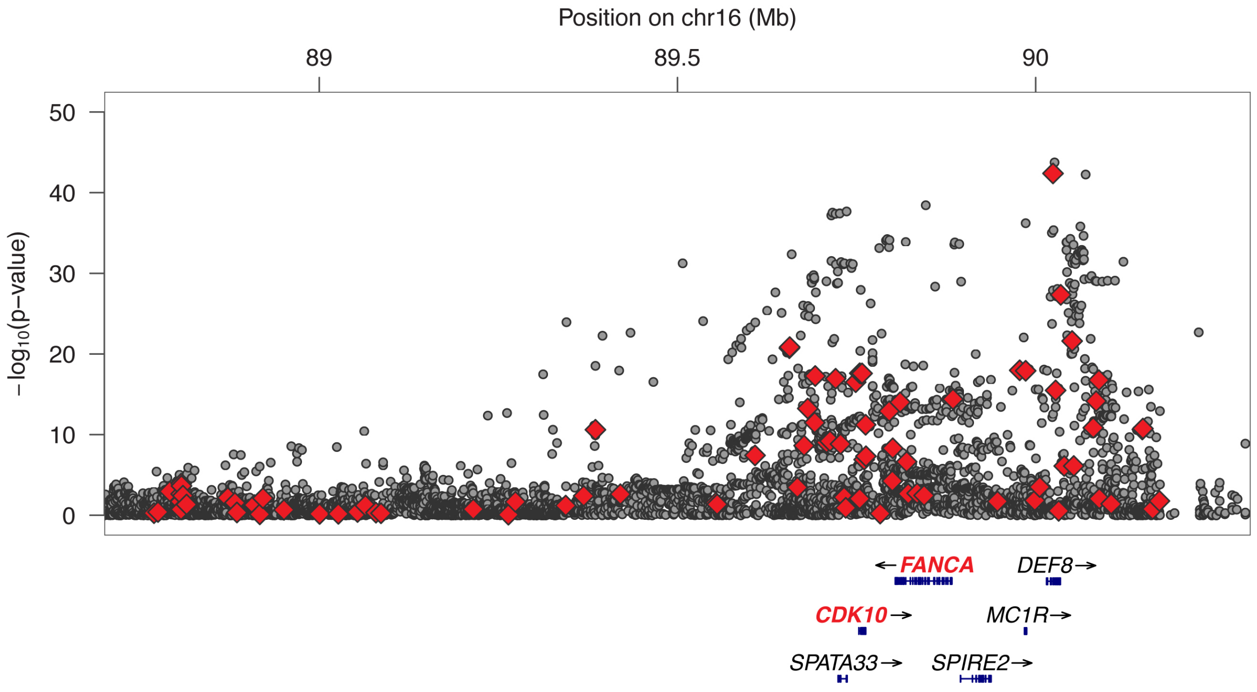

Manhattan plot of individual SNP cSCC associations at the 16q24 locus. Significance levels from the Kaiser cSCC GWAS[1] for all SNPs in the region (gray circles) and the subset of SNPs (red diamonds) with nonzero coefficients in one or more of the prediXcan expression models for the associated genes (red text), plotted as in Supplementary Fig. 6.

Supplementary Figure 9

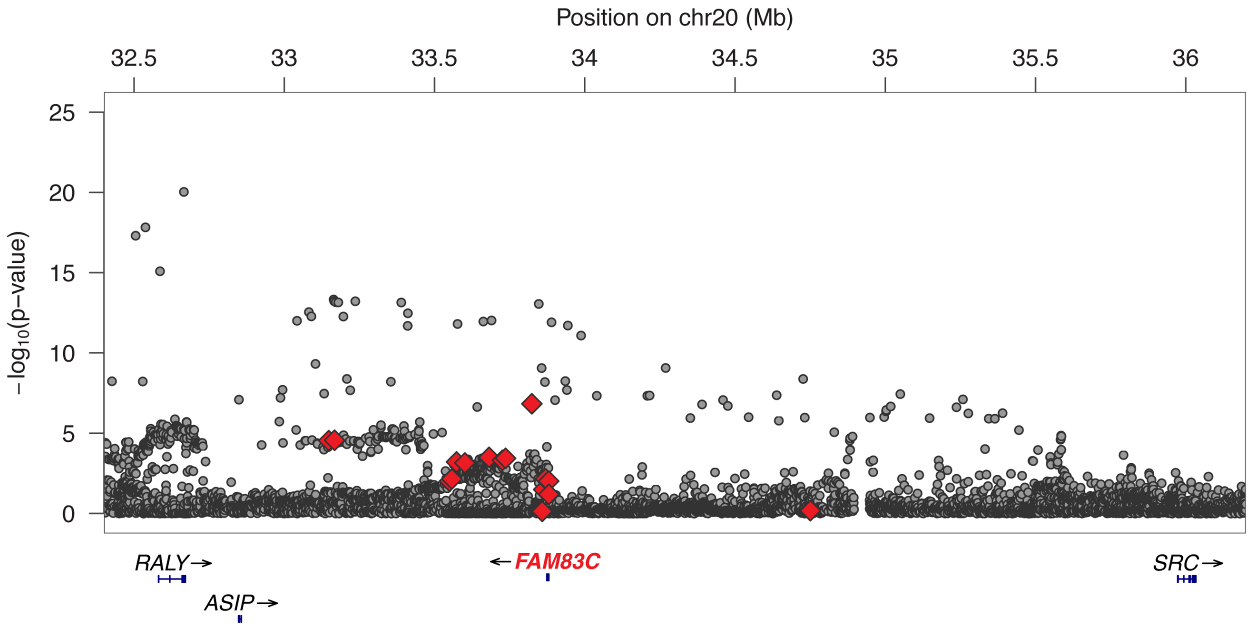

Manhattan plot of individual SNP cSCC associations at the 20q11 locus. Significance levels from the Kaiser cSCC GWAS[1] for all SNPs in the region (gray circles) and the subset of SNPs (red diamonds) with nonzero coefficients in the prediXcan expression model for the associated gene (red text), plotted as in Supplementary Fig. 6.

Supplementary Table 1
Sun-exposed skin associations with FDR < 10% in the Kaiser GERA cohort.

		Kaiser GERA analysis				23andMe analysis			
**Gene**	**Locus**	**FDR**	***P*-value**	**Beta**	**Beta SE**	**FDR**	***P*-value**	**Beta**	**Beta SE**
CDK10	16q24	1.90E-20	2.02E-23	-0.264	0.026	7.16E-57	4.77E-58	-0.212	0.013
HORMAD1	1q21	2.77E-02	8.59E-05	-0.086	0.022	1.27E-03	4.68E-04	-0.038	0.011
GOLPH3L	1q21	2.77E-02	8.83E-05	-0.094	0.024	1.27E-03	5.09E-04	-0.041	0.012
CASP8	2q33	4.18E-02	1.85E-04	-0.097	0.026	8.40E-08	1.12E-08	-0.073	0.013
CISD2	4q24	4.18E-02	2.22E-04	0.112	0.030	3.64E-01	2.91E-01	0.016	0.015
GSTO2	10q25	5.55E-02	3.64E-04	-0.083	0.023	8.42E-01	7.86E-01	0.003	0.011
MAPRE1	20q11	5.55E-02	5.31E-04	0.088	0.025	7.95E-03	4.24E-03	0.036	0.013
AHI1	6q23	5.55E-02	4.79E-04	0.077	0.022	6.19E-05	1.65E-05	0.046	0.011
ANXA9	1q21	5.55E-02	5.12E-04	-0.111	0.032	1.61E-03	7.51E-04	-0.053	0.016
HAL	12q23	6.33E-02	9.98E-04	-0.079	0.024	3.42E-07	6.83E-08	-0.064	0.012
SSBP4	19p13	6.33E-02	9.06E-04	-0.179	0.054	1.79E-01	1.31E-01	-0.037	0.024
SLC22A5	5q31	6.33E-02	1.01E-03	-0.086	0.026	8.42E-01	7.86E-01	-0.003	0.013
SKIV2L	6p21	6.33E-02	8.52E-04	-0.100	0.030	5.02E-02	3.01E-02	-0.031	0.015
C4A	6p21	6.33E-02	7.66E-04	-0.102	0.030	9.78E-01	9.78E-01	0.000	0.015
ACTR3	2q14	6.91E-02	1.17E-03	0.112	0.035	5.25E-02	3.50E-02	0.035	0.017

All genes in sun-exposed skin whose imputed expression levels were associated with cSCC at FDR < 10% in the Kaiser GERA cohort in the discovery phase, ordered by significance in the Kaiser GERA cohort. Their validation results in the 23andMe dataset are also shown.

P-value, from Wald test; Beta SE, standard error in effect size (beta).

Supplementary Table 2
Non-sun-exposed skin associations with FDR < 10% in the Kaiser GERA cohort.

		Kaiser GERA analysis				23andMe analysis			
Gene	Locus	FDR	*P*-value	Beta	Beta SE	FDR	*P*-value	Beta	Beta SE
CDK10	16q24	4.21E-31	5.57E-34	-0.281	0.023	6.53E-80	5.02E-81	-0.209	0.011
FAM83C	20q11	1.06E-02	2.80E-05	0.164	0.039	2.63E-12	4.05E-13	0.133	0.018
GSTO2	10q25	1.45E-02	5.74E-05	-0.096	0.024	7.98E-01	7.37E-01	0.004	0.012
HORMAD1	1q21	2.04E-02	1.08E-04	-0.084	0.022	4.03E-03	1.86E-03	-0.033	0.011
POU5F1	6p21	2.28E-02	1.51E-04	-0.100	0.026	4.36E-03	2.36E-03	-0.040	0.013
GOLPH3L	1q21	2.66E-02	2.11E-04	-0.104	0.028	4.36E-03	2.68E-03	-0.041	0.014
CASP8	2q33	2.78E-02	2.58E-04	-0.101	0.028	6.93E-07	1.60E-07	-0.071	0.014
MAPRE1	20q11	4.23E-02	4.48E-04	0.089	0.025	5.45E-02	4.19E-02	0.025	0.012
BTN3A2	6p22	4.53E-02	5.40E-04	-0.058	0.017	5.76E-01	4.87E-01	-0.006	0.008
AHI1	6q23	4.98E-02	7.25E-04	0.076	0.022	4.52E-05	1.74E-05	0.047	0.011
TMEM230	20p12	7.71E-02	1.43E-03	0.119	0.037	8.59E-01	8.59E-01	0.003	0.018
SLC35F5	2q14	7.71E-02	1.34E-03	0.146	0.045	1.07E-02	7.43E-03	0.060	0.022
HAL	12q23	7.72E-02	1.53E-03	-0.085	0.027	1.83E-06	5.63E-07	-0.066	0.013

All genes in non-sun-exposed skin whose imputed expression levels were associated with cSCC at FDR < 10% in the Kaiser GERA cohort in the discovery phase, ordered by significance in the Kaiser GERA cohort. Their validation results in the 23andMe dataset are also shown.

P-value, from Wald test; Beta SE, standard error in effect size (beta).

Supplementary Table 3
LCL associations with FDR < 10% in the Kaiser GERA cohort.

		Kaiser GERA analysis				23andMe analysis			
**Gene**	**Locus**	**FDR**	***P*-value**	**Beta**	**Beta SE**	**FDR**	***P*-value**	**Beta**	**Beta SE**
CDK10	16q24	1.10E-19	1.71E-22	-0.295	0.030	6.58E-62	6.58E-63	-0.245	0.015
FANCA	16q24	2.31E-08	7.19E-11	-0.238	0.037	2.06E-30	4.12E-31	-0.201	0.017
CTSS	1q21	2.07E-03	9.68E-06	-0.106	0.024	1.62E-07	4.85E-08	-0.065	0.012
C4A	6p21	6.48E-03	4.04E-05	-0.147	0.036	8.45E-01	8.45E-01	-0.004	0.018
HLA-DQB1	6p21	1.83E-02	1.42E-04	-0.059	0.016	8.45E-01	8.21E-01	0.002	0.009
ORMDL3	17q21	2.67E-02	2.50E-04	0.081	0.022	1.90E-03	9.49E-04	0.036	0.011
C9orf69	9q34	8.26E-02	9.01E-04	-0.085	0.025	8.45E-01	8.34E-01	0.002	0.009
GSDMB	17q21	9.08E-02	1.27E-03	0.108	0.034	3.70E-03	2.22E-03	0.050	0.016
AHI1	6q23	9.08E-02	1.24E-03	0.085	0.026	6.40E-04	2.56E-04	0.046	0.013
LEKR1	3q25	9.10E-02	1.42E-03	0.100	0.031	1.67E-02	1.17E-02	0.038	0.015

All genes in LCLs whose imputed expression levels were associated with cSCC at FDR < 10% in the Kaiser GERA cohort in the discovery phase, ordered by significance in the Kaiser GERA cohort. Their validation results in the 23andMe dataset are also shown.

P-value, from Wald test; Beta SE, standard error in effect size (beta).

Supplementary Table 4
Whole blood associations with FDR < 10% in the Kaiser GERA cohort.

		Kaiser GERA analysis				23andMe analysis			
**Gene**	**Locus**	**FDR**	***P*-value**	**Beta**	**Beta SE**	**FDR**	***P*-value**	**Beta**	**Beta SE**
CDK10	16q24	7.35E-19	9.09E-22	-0.213	0.022	1.37E-59	1.14E-60	-0.176	0.011
SKIV2L	6p21	3.32E-03	8.20E-06	-0.143	0.032	1.07E-03	3.58E-04	-0.056	0.016
C4A	6p21	5.99E-03	2.22E-05	-0.120	0.028	2.17E-01	1.63E-01	-0.020	0.014
HLA-DRB5	6p21	8.16E-03	4.03E-05	-0.081	0.020	2.40E-03	9.98E-04	-0.037	0.011
ORMDL3	17q21	1.24E-02	7.64E-05	0.113	0.029	4.44E-03	2.57E-03	0.042	0.014
GSDMB	17q21	1.76E-02	1.42E-04	0.113	0.030	4.44E-03	2.59E-03	0.044	0.015
HERC2	15q13	1.76E-02	1.52E-04	-0.106	0.028	1.61E-11	2.69E-12	-0.097	0.014
CTSH	15q25	3.32E-02	4.05E-04	-0.098	0.028	1.50E-01	1.00E-01	-0.023	0.014
SLC22A18	11p15	3.32E-02	4.11E-04	0.121	0.034	8.73E-01	8.73E-01	-0.002	0.012
HLA-DOB	6p21	3.32E-02	3.77E-04	-0.079	0.022	1.35E-05	3.38E-06	-0.051	0.011
SLC22A5	5q31	5.28E-02	7.18E-04	-0.125	0.037	8.73E-01	8.56E-01	-0.003	0.018
C4B	6p21	6.68E-02	9.91E-04	0.089	0.027	3.40E-01	2.83E-01	-0.015	0.014

All genes in whole blood whose imputed expression levels were associated with cSCC at FDR < 10% in the Kaiser GERA cohort in the discovery phase, ordered by significance in the Kaiser GERA cohort. Their validation results in the 23andMe dataset are also shown.

P-value, from Wald test; Beta SE, standard error in effect size (beta).

Supplementary Table 5
Expression level correlations in the 1q21 locus.

Gene	*CTSS* (LCLs)	*HORMAD1* (SE skin)	*GOLPH3L* (SE skin)	*ANXA9* (SE skin)
CTSS (LCLs)	1.0 † [51 *]	0.823 [10]	0.843 [9]	0.712 [1]
HORMAD1 (SE skin)		1.0 [33]	0.991 [25]	0.714 [4]
GOLPH3L (SE skin)			1.0 [64]	0.699 [1]
ANXA9 (SE skin)				1.0 [27]

† Pearson correlation coefficients between imputed expression levels of the indicated pair of genes among individuals in the Kaiser GERA cohort.
* Numbers of overlapping SNPs [in brackets] in the prediXcan expression imputation models for the indicated pair of genes.

SE skin, sun-exposed skin.

Supplementary Table 6
Adjusted logistic regressions in the 1q21 locus.

**Covariate(s) in model †**	***P*-value**	**Beta**
CTSS (LCLs)	9.68E-06	-0.106
HORMAD1 (SE skin)	8.59E-05	-0.086
GOLPH3L (SE skin)	8.83E-05	-0.094
ANXA9 (SE skin)	5.12E-04	-0.111
CTSS (LCLs)	3.65E-02	-0.087
HORMAD1 (SE skin)	6.00E-01	-0.020
CTSS (LCLs)	3.80E-02	-0.091
GOLPH3L (SE skin)	6.99E-01	-0.017
CTSS (LCLs)	5.31E-03	-0.094
ANXA9 (SE skin)	6.45E-01	-0.021
HORMAD1 (SE skin)	7.53E-01	-0.051
GOLPH3L (SE skin)	8.28E-01	-0.039
HORMAD1 (SE skin)	3.66E-02	-0.065
ANXA9 (SE skin)	3.43E-01	-0.043
GOLPH3L (SE skin)	3.48E-02	-0.071
ANXA9 (SE skin)	3.08E-01	-0.046
CTSS (LCLs)	5.48E-02	-0.092
HORMAD1 (SE skin)	5.83E-01	-0.094
GOLPH3L (SE skin)	6.40E-01	0.091
ANXA9 (SE skin)	8.44E-01	-0.010

Logistic regressions of cSCC case/control status against one or more imputed gene expression levels (as shown) performed in the Kaiser GERA cohort.

† Additional covariates included sex, age, and ten ancestry principal components (not shown), as described in Methods.

SE skin, sun-exposed skin.

Supplementary Table 7
Expression level and dosage correlations in the 6p21 locus.

Gene	*HLA-DOB* (whole blood)	*SKIV2L* (whole blood)	*HLA-DRB5* (whole blood)	rs4455710	GWAS SNPs **
HLA-DOB (whole blood)	1.0 † [33 *]	0.052 [0]	0.044 [1]	-0.258	0
SKIV2L (whole blood)		1.0 [29]	0.097 [0]	-0.332	0
HLA-DRB5 (whole blood)			1.0 [24]	-0.511	1

† Pearson correlation coefficients between imputed expression levels of the indicated pair of genes and/or SNP dosages among individuals in the Kaiser GERA cohort.
* Numbers of overlapping SNPs [in brackets] in the prediXcan expression imputation models for the indicated pair of genes.
** Number of SNPs in the prediXcan expression imputation model for the indicated gene that also met genome-wide significance in the original Kaiser cSCC GWAS[1].

Supplementary Table 8
Adjusted logistic regressions in the 6p21 locus.

Covariate(s) in model †	*P*-value	Beta
HLA-DOB (whole blood)	3.77E-04	-0.079
SKIV2L (whole blood)	8.20E-06	-0.143
HLA-DRB5 (whole blood)	4.03E-05	-0.081
HLA-DOB (whole blood)	9.17E-04	-0.074
SKIV2L (whole blood)	1.94E-05	-0.137
HLA-DOB (whole blood)	8.22E-04	-0.074
HLA-DRB5 (whole blood)	8.68E-05	-0.078
SKIV2L (whole blood)	4.80E-05	-0.131
HLA-DRB5 (whole blood)	2.43E-04	-0.073
HLA-DOB (whole blood)	1.63E-03	-0.070
SKIV2L (whole blood)	9.35E-05	-0.126
HLA-DRB5 (whole blood)	4.31E-04	-0.070
HLA-DOB (whole blood)	1.98E-01	-0.030
rs4455710	2.05E-16	0.167
SKIV2L (whole blood)	1.11E-01	-0.054
rs4455710	5.82E-15	0.163
HLA-DRB5 (whole blood)	6.32E-01	0.011
rs4455710	4.45E-15	0.180
rs4455710	6.47E-19	0.174

Logistic regressions of cSCC case/control status against one or more imputed gene expression levels and/or SNP dosages (as shown) performed in the Kaiser GERA cohort.

† Additional covariates included sex, age, and ten ancestry principal components (not shown), as described in Methods.

Supplementary Table 9
Expression level and dosage correlations in the 16q24 locus.

Gene	*CDK10* (NSE skin)	*CDK10* (SE skin)	*CDK10* (LCLs)	*CDK10* (whole blood)	*FANCA* (LCLs)	rs4268748	GWAS SNPs **
CDK10 (NSE skin)	1.0 † [35 *]	0.947 [8]	0.871 [2]	0.891 [6]	0.189 [2]	-0.708	16
CDK10 (SE skin)		1.0 [16]	0.958 [3]	0.963 [7]	0.041 [0]	-0.612	8
CDK10 (LCLs)			1.0 [16]	0.974 [3]	-0.060 [0]	-0.558	9
CDK10 (whole blood)				1.0 [32]	-0.010 [1]	-0.564	12
FANCA (LCLs)					1.0 [19]	-0.258	5

† Pearson correlation coefficients between imputed expression levels of the indicated pair of genes and/or SNP dosages among individuals in the Kaiser GERA cohort.
* Numbers of overlapping SNPs [in brackets] in the prediXcan expression imputation models for the indicated pair of genes.
** Number of SNPs in the prediXcan expression imputation model for the indicated gene that also met genome-wide significance in the original Kaiser cSCC GWAS[1].

SE skin, sun-exposed skin; NSE skin, non-sun exposed skin.

Supplementary Table 10
Adjusted logistic regressions in the 16q24 locus.

Covariate(s) in model †	*P*-value	Beta
CDK10 (NSE skin)	5.57E-34	-0.281
CDK10 (SE skin)	2.02E-23	-0.264
CDK10 (LCLs)	1.71E-22	-0.295
CDK10 (whole blood)	9.09E-22	-0.213
FANCA (LCLs)	7.19E-11	-0.238
CDK10 (NSE skin)	3.85E-22	-0.794
CDK10 (SE skin)	5.20E-09	1.005
CDK10 (LCLs)	5.36E-05	-0.658
CDK10 (whole blood)	2.81E-01	0.129
CDK10 (NSE skin)	2.42E-29	-0.265
FANCA (LCLs)	4.61E-06	-0.170
CDK10 (SE skin)	5.20E-23	-0.263
FANCA (LCLs)	1.88E-10	-0.234
CDK10 (LCLs)	4.59E-25	-0.316
FANCA (LCLs)	2.22E-13	-0.270
CDK10 (whole blood)	8.90E-23	-0.221
FANCA (LCLs)	7.44E-12	-0.252
CDK10 (NSE skin)	2.55E-03	-0.100
rs4268748	1.72E-14	0.252
CDK10 (SE skin)	1.07E-01	-0.054
rs4268748	5.83E-24	0.294
CDK10 (LCLs)	3.27E-02	-0.078
rs4268748	1.23E-25	0.290
CDK10 (whole blood)	6.39E-02	-0.050
rs4268748	5.04E-26	0.294
FANCA (LCLs)	2.50E-03	-0.115
rs4268748	1.39E-37	0.305
rs4268748	5.04E-45	0.323

Logistic regressions of cSCC case/control status against one or more imputed gene expression levels and/or SNP dosages (as shown) performed in the Kaiser GERA cohort.

† Additional covariates included sex, age, and ten ancestry principal components (not shown).

Supplementary Table 11
Kaiser TWAS results with Bonferroni correction.

			PrediXcan	Kaiser GERA analysis		
**Locus**	**Gene**	**Tissue**	R^2	***P*-value**	**Beta**	**Beta SE**
1q21	*CTSS*	LCLs	0.397	9.68E-06	-0.106	0.024
6p21	*SKIV2L*	Whole blood	0.259	8.20E-06	-0.143	0.032
	C4A	Whole blood	0.205	2.22E-05	-0.120	0.028
		LCLs	0.229	4.04E-05	-0.147	0.036
	HLA-DRB5	Whole blood	0.592	4.03E-05	-0.081	0.020
10q25	*GSTO2*	Skin (non-sun-exp)	0.301	5.74E-05	-0.096	0.024
16q24	*CDK10*	Skin (non-sun-exp)	0.383	5.57E-34	-0.281	0.023
		Skin (sun-exp)	0.396	2.02E-23	-0.264	0.026
		LCLs	0.208	1.71E-22	-0.295	0.030
		Whole blood	0.423	9.09E-22	-0.213	0.022
	FANCA	LCLs	0.207	7.19E-11	-0.238	0.037
20q11	*FAM83C*	Skin (non-sun-exp)	0.215	2.80E-05	0.164	0.039

Results of a TWAS performed with a Bonferroni-corrected significance threshold in the Kaiser GERA cohort, for comparison with the previous Kaiser GWAS[1] and with the discovery-validation approach used in the main text.

R^2, squared correlation coefficient for the prediXcan imputation model; *P*-value, from Wald test; Beta SE, standard error in effect size (beta); exp, exposed.

Supplementary References

1. Asgari, M.M. *et al.* Identification of susceptibility loci for cutaneous squamous cell carcinoma. *J Invest Dermatol* **136**, 930-7 (2016).

2. Pruim, R.J. *et al.* LocusZoom: regional visualization of genome-wide association scan results. *Bioinformatics* **26**, 2336-7 (2010).
